# Upcycling of surgical facemasks into carbon based thin film electrode for supercapacitor technology

**DOI:** 10.1038/s41598-023-37499-x

**Published:** 2023-07-27

**Authors:** Aamir Ahmed, Sonali Verma, Prerna Mahajan, Ashok K. Sundramoorthy, Sandeep Arya

**Affiliations:** 1 Department of Physics, University of Jammu, Jammu, Jammu and Kashmir 180006 India; 2 Centre for Nano-Biosensors, Department of Prosthodontics, Saveetha Dental College and Hospitals, Saveetha Institute of Medical and Technical Sciences, Chennai, Tamil Nadu 600077 India

**Keywords:** Energy science and technology, Materials science

## Abstract

Polypropylene (PP), a commonly used plastic, is used for making the outer layers of a surgical face mask. In 2020, around 3 billion surgical face masks were disposed into the environment, causing a huge threat to wildlife, aquatic life, and ecosystems. In this work, we have reported the sulfonation technique for stabilizing the surgical face masks and their conversion into carbon nanoparticles for application as a supercapacitor electrode. The electrode is fabricated by preparing a slurry paste of carbon nanoparticles and pasting it on a conductive wearable fabric. To investigate the performance of the carbon thin film electrode, electrochemical techniques are employed. The Cyclic Voltammetry (CV) analysis performed at different scan rates in a 6 molar KOH electrolyte reveals that the carbon thin film acts as a positive electrode. At 4 A g^−1^, the electrode shows a specific capacitance of 366.22 F g^−1^ and 100% retention of specific capacitance for 8000 cycles. A two-electrode asymmetric device is fabricated using carbon thin film as the positive electrode, NiO thin film as the negative electrode, and a KOH separator between two electrodes. The device shows a specific capacitance of 113.73 F g^−1^ at 1.3 A g^−1^ and glows a red LED for 6 min. This work is a step towards upcycling the waste produced from surgical face masks used during the COVID-19 pandemic and its application for energy storage.

## Introduction

The waste material produced by households and industries causes detrimental damage to the environment, wildlife, aquatic animals, and human health^1^. The toxic materials present in these wastes affect the living organisms in soil, water, and air. However, recent developments have led to the exploitation of these waste materials into useful resources^2^. In 2019, the COVID-19 pandemic struck the world and one of the precautionary measures was using surgical face masks. This led to a massive surge in the production of face masks around the globe^3–6^. The surgical face masks are single-use materials made from polypropylene (PP). The PP is a type of plastic and extensive consumption of these masks has added to the issue of global plastic waste^7^. According to an estimate by National Geographic Magazine, in 2020, more than 3 billion masks were disposed of daily and the trend persisted for the following 2 years^8^. All these PP-based surgical face masks when dumped into the environment cause serious threats to ecosystems. Thus, there is a need to address this issue and find effective solutions for recycling or upcycling these used face masks for sustainable development. One such solution is their conversion into carbon-based nanomaterials (CNMs). The most commonly used CNMs are graphene, activated carbon, and carbon nanotubes (CNTs) and their production from wastes has various potential applications. CNMs have extremely good morphological, chemical, mechanical, and electrical properties which significantly increase their application for developing sensors, supercapacitors, transistors, photo-electric devices, etc.^9^. Moreover, CNMs are produced easily, efficiently, and cost-effectively using various synthesis techniques^10,11^. Polypropylene (PP) has been reduced to CNMs via various chemical methods^12,13^. The production of CNMs from wastes (masks) will significantly reduce the quantity of these wastes in the environment and help to clean the environment and ecosystems.

In this work, we have reported the sulfonation technique for stabilizing the face masks and their conversion into carbon nanoparticles for application as a supercapacitor electrode. The standard characterization tools are used to examine the morphology, constituent elements, and functional groups present in the synthesized materials. The electrochemical performance of the fabricated electrodes and their supercapacitor application is analyzed using CV, Galvanostatic Charge or Discharge (GCD), and Electrochemical Impedance Spectroscopy (EIS). The electrodes are fabricated on a wearable cotton fabric. The carbon thin film electrode shows good electrochemical performance i.e., specific capacitance, cyclic stability, capacitance retention, etc. This work is a step towards upcycling the waste produced from the facial masks used during the COVID-19 pandemic and its application in supercapacitor technology. The research will aid in the development of energy storage devices as well as environmental remediation. The carbon thin film electrode reported depicts good capacitive behavior in comparison to some previously published works.

## Experiment

### Chemicals

The chemical and materials used during the synthesis procedure were surgical face masks, sulphuric acid (H_2_SO_4_) (98%, SDFCL), potassium hydroxide (KOH) (97%, RANKEM), nickel nitrate hexahydrate (Ni(NO_3_)_2_·6H_2_O) (98%, Alfa Aesar), ammonium hydroxide (NH_4_OH) (25%, Emplura), *N*-Methyl-2-pyrrolidone (NMP) (99.9%, Spectrochem), polyvinyl alcohol (PVA) (99%, Sigma-Aldrich), ethanol, and DI (De-ionized) water. The chemicals were used as received. However, the surgical masks were cleaned using ethanol and DI water before their application.

### Synthesis of carbon nanoparticles from surgical face masks

**Figure 1:**
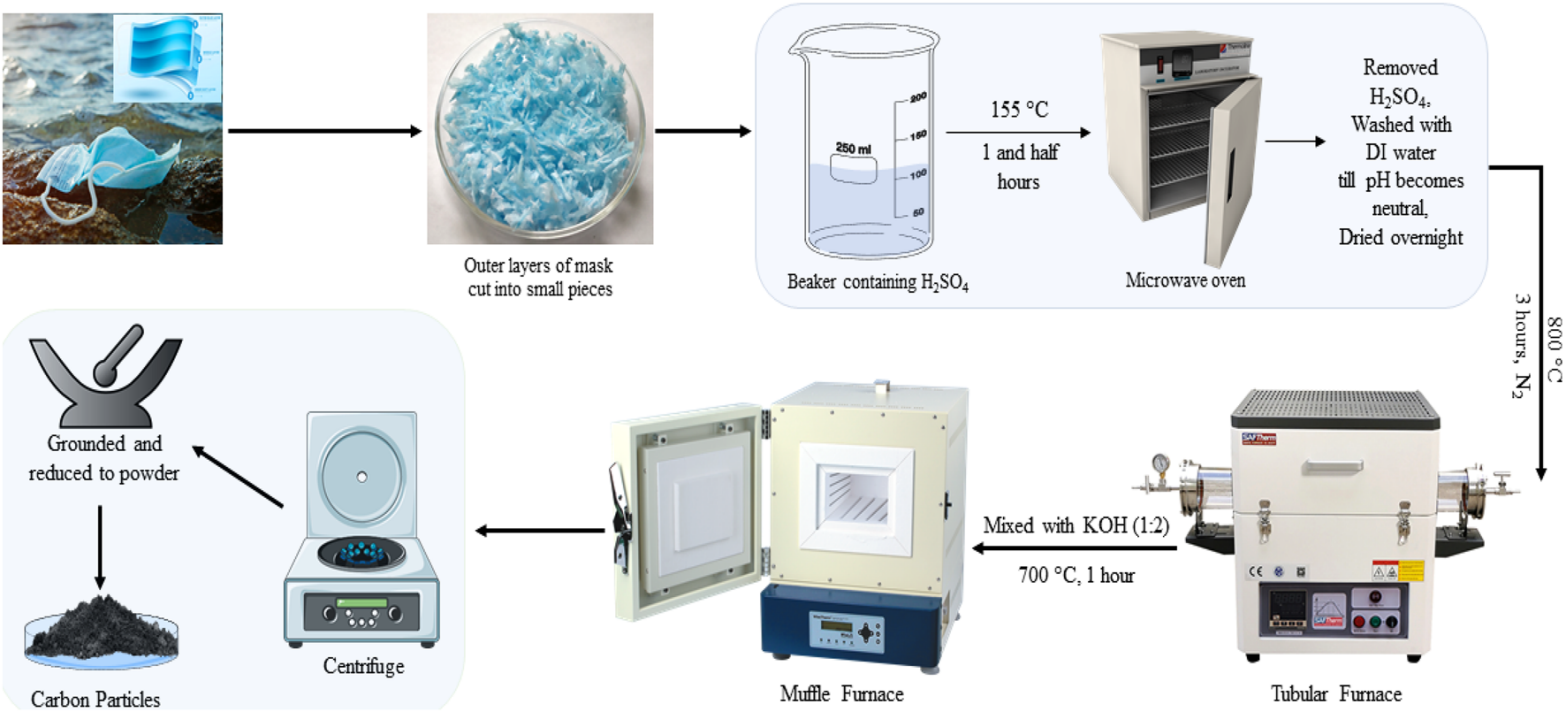
Step-by-step synthesis route of carbon nanoparticles.

The two outer layers of a surgical face mask are used during the synthesis. The outer layers of a face mask are made of polypropylene, which is a good source of carbon. The outer layers were cut into small pieces (1 g) and put into 25 mL of concentrated H_2_SO_4_ in a glass beaker. The beaker was then placed in a muffle furnace and heated at 155 °C for 1.5 h. After heating, the sulphuric acid was removed from the beaker, and the sample (black mass) was obtained. The sample was washed with DI water till its pH becomes neutral. The sample was then dried overnight and again heated for 3 h at a temperature of 800 °C in the presence of nitrogen. The sample now obtained was ground with KOH in a 1:2 ratio and heated at 700 °C for 1 h. The final obtained material was then centrifuged, dried, and grounded to produce carbon powder. Figure 1 diagrammatically represents the step-by-step synthesis procedure.

### Fabrication of carbon-based thin film electrode

The conductive cotton fabric (CF) is used as a flexible substrate for fabricating electrodes (Fig. Sa under supplementary information). The as-synthesized carbon powder is mixed with PVA in a ratio of 1:9 in a beaker. To this mixture, NMP solvent was added drop-wise, heated and stirred continuously until a uniform and slurry black paste is obtained. The paste is then applied over CF and heated at 95 °C to form the final carbon electrode. The fabricated carbon electrode is shown in Fig. Sb (under supplementary information).

### Fabrication of nickel oxide (NiO) thin film electrode

The sol–gel method is an easy and cheap method used for the synthesis of nanoparticles. A 2.0 M solution of ammonium hydroxide (NH_4_OH) in DI water is poured drop-wise into a 0.5 M solution of nickel nitrate hexahydrate in DI water. The solution mixture is stirred at 100 °C for 4 h and left for 24 h aging. The sample is centrifuged at 7000 rpm using DI water and ethanol, annealed for 2 h at a temperature of 200 °C. Finally, the sample is ground and reduced to light-green NiO nanoparticles.

For preparing the NiO electrode, a slurry paste is prepared by mixing NiO nanoparticles, graphite powder, and PVA in a ratio of 8:1:1. The NMP is added dropwise into this mixture and continuously heated until a slurry paste is obtained. The prepared NiO paste is then applied on CF and heated on the hot plate to form NiO thin film electrode (Fig. Sc under supplementary information).

## Results and discussion

### Morphology and chemical composition

#### Carbon nanoparticles

**Figure 2:**
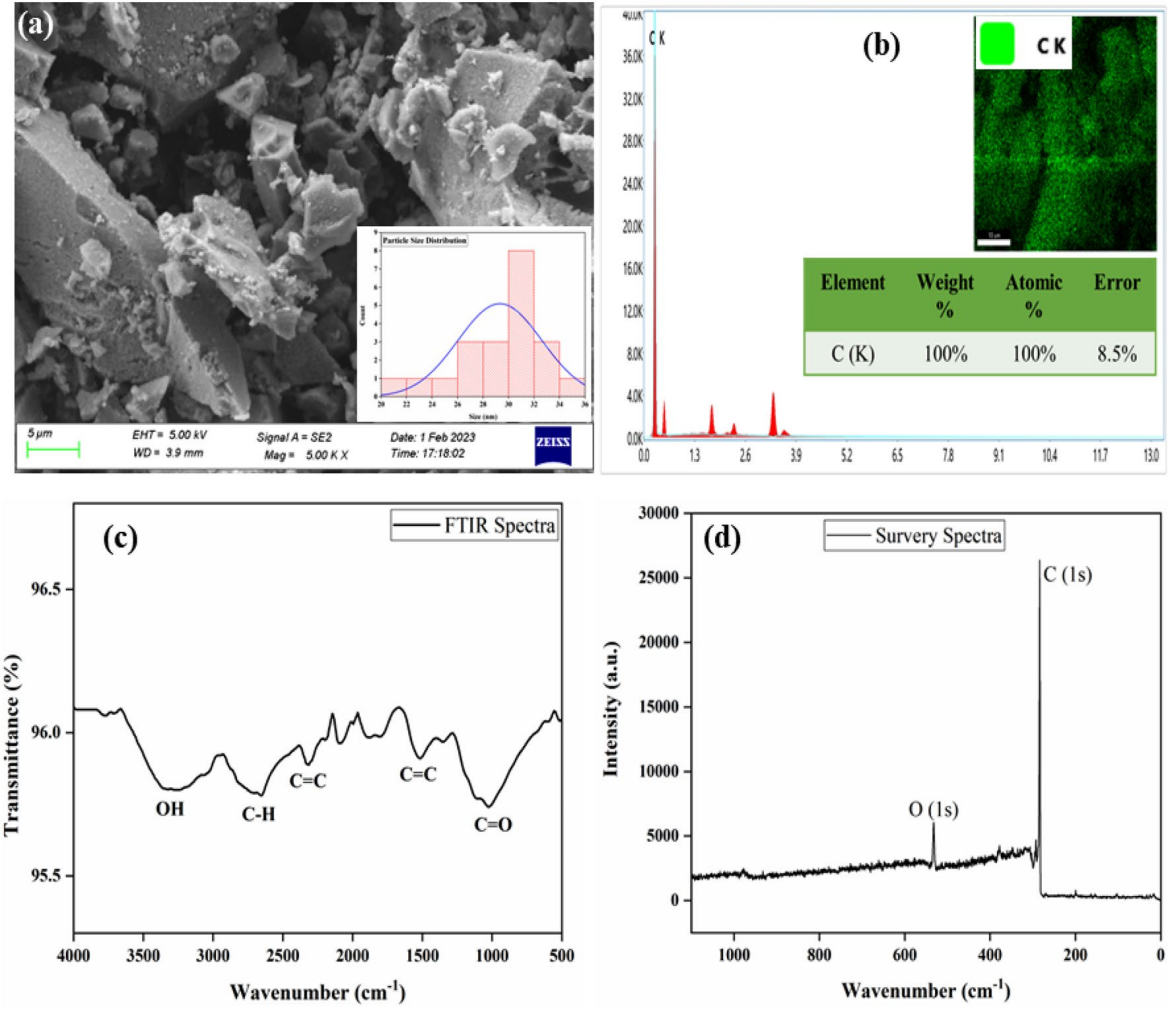
(**a**) FESEM image, (**b**) EDS spectra and mapping, (**c**) FTIR spectra, and (**d**) XPS spectra of the synthesized carbon.

The morphology and chemical composition of the produced carbon is analyzed using standard characterization tools. The FESEM image shown in Fig. 2a depicts the surface morphology of the nanoparticles along with the particle size distribution. Using ImageJ software, the average size of the carbon particles is found to be 29 nm. Nanomaterials have been used extensively for the fabrication of supercapacitor electrodes. The nanomaterials show high specific capacitance and good electrochemical performance, as they offer more surface area for energy storage^14^. Moreover, the nanomaterials possess good mechanical properties which help in attaining high coulomb efficiency and mechanical integrity while performing the cyclic stability tests^15^. The carbon nanoparticles are in the form of flakes and possess smooth morphology. Figure 2b shows the EDS spectra along with the elemental mapping and weight % of the nanoparticles. The EDS spectra show a peak with maximum intensity for C(K). The elemental mapping along with atomic and weight% ratios further clarify the synthesis of carbon nanoparticles.

FTIR technique is a useful analytical method for the identification of constituents and helps in investigating the functional groups present in the material. Figure 2c is the FTIR spectra of the carbon. The broadband between 3100 and 3400 cm^−1^ signifies the presence of the -OH group of water molecules (moisture) whereas the band at 2500–3000 cm^−1^ indicates the presence of an aliphatic –CH stretching^16,17^. The transitions at 2316 and 1510 cm^−1^ indicate the C=C stretching whereas the peak at 2090 cm^−1^ is due to the interaction of sulphur with the face mask developed during the sulphonation process^18^. The peak at 1029 cm^−1^ denotes stretching vibrations of C=O^19^. The XPS survey spectra in Fig. 2d clearly show the standard peaks for C(1*s*) and O(1*s*) at 284.4 and 532.4 eV, respectively. Moreover, the high-resolution spectra shown in Fig. S1 (supplementary information) indicate that the peak for C(1*s*) is for the *sp*^2^ hybridized C=C, C–C, and C=O bonds^20^. Thus, XPS further confirms the results obtained from FTIR spectra. Hence, the FESEM, EDS, FTIR, and XPS studies confirm the formation of carbon nanoparticles.

#### NiO nanoparticles

**Figure 3:**
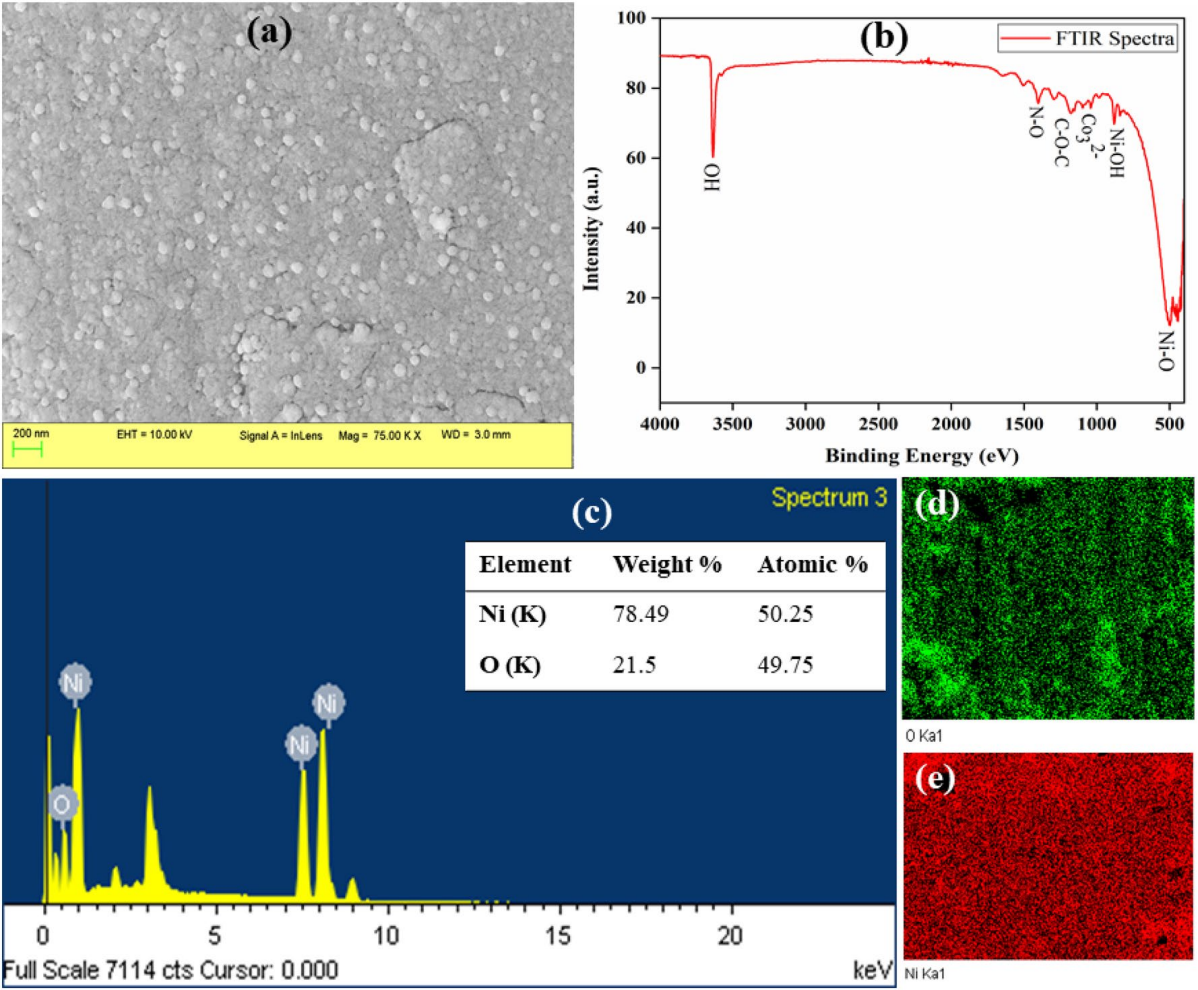
(**a**) FESEM image, (**b**) FTIR spectra, and (**c**) EDS spectra of NiO nanoparticles along with (**d**) mapping of O, and (**e**) mapping of Ni.

The FESEM image of the NiO nanoparticles as shown in Fig. 3a indicates that the particles possess uniform distribution and are spherical. The morphology of the nanoparticles i.e., shape, size, surface area, etc. plays a crucial role in determining the electrochemical performance of an electrode^21–23^. The FTIR spectra in Fig. 3b show different peaks. The transition at 3636 cm^−1^ is for the –OH bond vibrations whereas the peak at 1401 cm^−1^ denotes N–O stretching mode or adsorbed NO^−^ produced from the precursors^24^. The C–O–C bond vibrations and adsorbed CO_3_^2−^ ions (due to adsorbed CO_2_) are denoted by the transitions at 1173 cm^−1^ and 1048 cm^−1^, respectively. Further, the peaks at 878 cm^−1^ and 500 cm^−1^ denote Ni–OH group vibrations and Ni–O stretching vibrations. Thus, FTIR results indicate the synthesis of NiO nanoparticles. Moreover, the EDS spectra in Fig. 3c show peaks for the Ni and O along with their weight and atomic percentages. The elemental mapping of the nanoparticles in Fig. 3d and e further confirms the synthesis of NiO nanoparticles.

### Electrochemical characterizations

The CHI600E instrument is used during the electrochemical study of the fabricated thin film electrodes. The three-electrode configuration is applied to perform the electrochemical analysis using Saturated Calomel Electrode (SCE) as the reference electrode, fabricated thin film as the working electrode, and platinum (Pt) counter electrode. The electrolyte is a 6.0 M solution of KOH in DI water and all the electrochemical characterizations are carried at room temperature. Using CV and GCD curves, the specific capacitance is calculated according to equations^25,26^:

1 $$ C = \frac{Area}{{nm\Delta VVs}} $$

2 $$ C = I \times \frac{\Delta t}{{m \times \Delta V}} $$

where, “*C*” corresponds to the specific capacitance calculated in Farad gram^−1^ (F g^−1^), “*n* = 1 or 2 for asymmetric and symmetric supercapacitor, “V_s_” denotes scan rate, “*ΔV”* denotes potential range, “*I*” is the discharge current in Amperes (*A*), “*Δt*” denotes discharging time in seconds (*s*), and “*m*” is the mass of the active electrode material. The “*Area*” in Eq. (1) is the area under the CV curve at a particular scan rate.

The energy density and power density of the electrodes are calculated using the following equations^26^:

3 $$ E = \frac{1}{2}C(\Delta V)^{2} $$

4 $$ P = \frac{E}{\Delta t} $$

where “*E*” and “*P*” denote energy and power density.

#### Carbon thin film electrode

**Figure 4:**
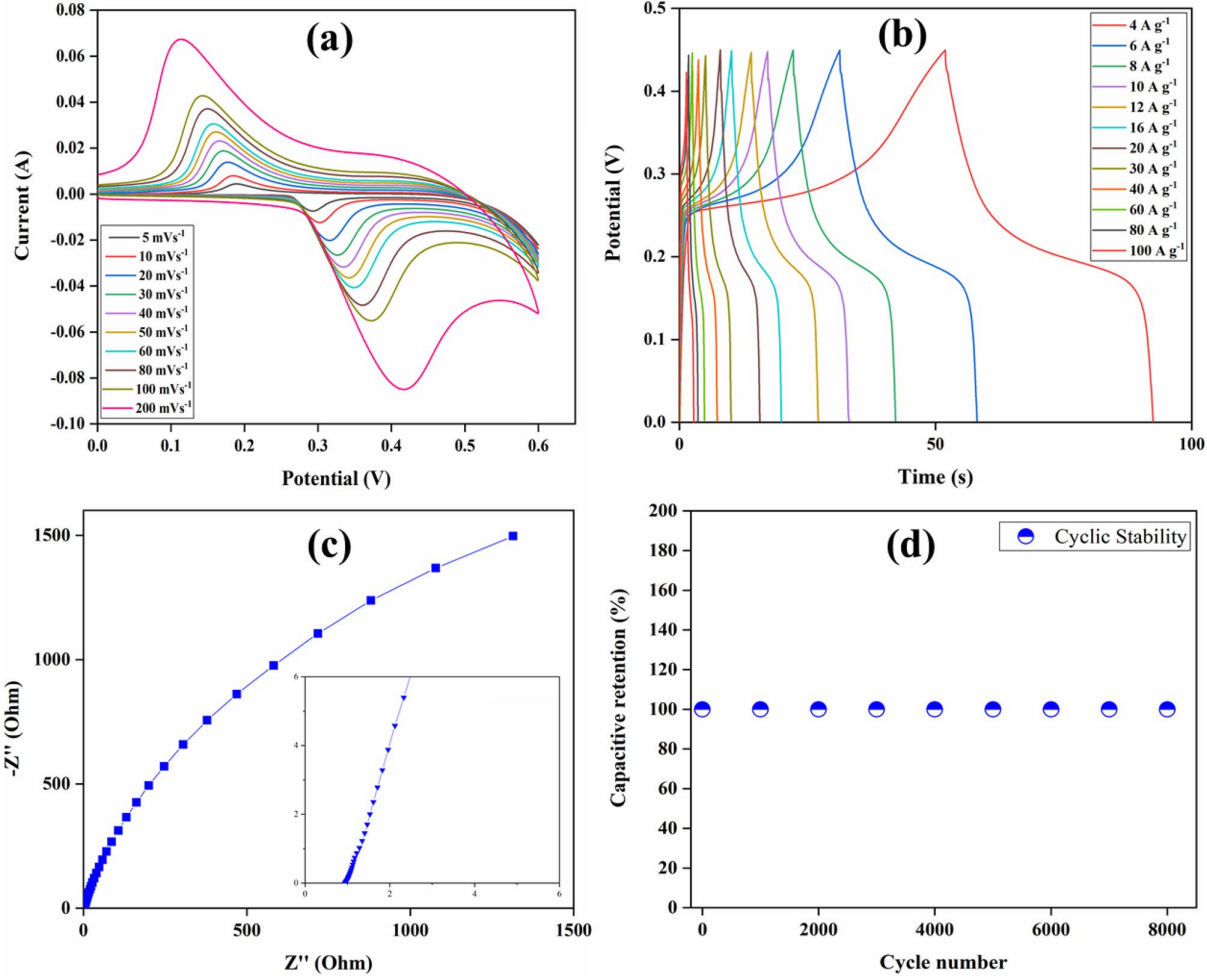
(**a**) CV, (**b**) GCD, (**c**) EIS, and (**d**) cyclic stability of the carbon thin film electrode.

Figure 4a represents the CV curves of the fabricated carbon thin film electrode. The CV measurements are carried at 5 to 200 mV s^−1^ scan rate over 0 to 0.6 V of the potential range. In each CV curve, there is a prominent redox peak which suggests that the capacitance of the thin film electrode follows the redox mechanism. The shape of the CV profiles of carbon electrode displayed faradaic-dominated CV profiles which can be classified as Type C curves^25,27^. The oxidation–reduction peaks are due to the redox reaction by the presence of structural defects and residual functional groups (–OH, COOH, etc.) with electrolyte ions^28^. Moreover, the peak current increases linearly with an increase in the scan rate, and the area under the curve also increases. This suggests the highly capacitive nature of electrode along with rapid electron/ion transport and interfacial faradic redox reactions. The symmetrical nature of the CV curves also suggests good electron and ion conduction of the electrode. Since the CV curves are only present in the positive potential region, the carbon thin film electrode can be used as a positive electrode for the supercapacitor application. Table S1 (supplementary information) represents the specific capacitance of the carbon thin film electrode calculated from the CV curves using Eq. (1).

The relation between peak current (*i*_*p*_) and scan rate (*v*) is determined according to Eq. (4) to understand the behavior of the electrode^26^.

5 $$ ip = av^{b} $$

where “*i*_*p*_” denotes the peak current in amperes, “*v*” is the scan rate in mV s^−1^, and “*a*” and “*b*” are variable parameters. The parameter “*b*” serves as the slope of the curve between *log i*_*p*_ and *log v*. For the surface-controlled process, the value of *b* = 1, and for the battery-type behavior, *b* = 0.5. The value of *b* is 0.74 for anodic peak currents and 0.65 for cathodic peak currents, signifying a diffusion-controlled process (Fig. S2 (supplementary information))^29^. In addition to this, Dunn’s method is used to study the charge storage mechanism of the electrode. Figure S3 (supplementary information) shows that the storage is mostly controlled by the diffusion process and the result is in agreement with the value of *“b”* calculated for the electrode.

At various current density, the GCD test performed for the electrode in the potential range of 0 to 0.45 V is shown in Fig. 4b. Using *m* = 0.0005 g in Eq. (1), the obtained specific capacitance values are 366.22, 361.33, 360.88, 351.11, 346.66, 348.44, 337.77, 326.66, 320, 306.66, 302.22, and 288.88 F g^-1^ at 4, 6, 8 10, 12, 16, 20, 30, 40, 60, 80, and 100 A g^−1^, respectively. The ions of the electrolyte diffuse easily into the electrode at low current density, due to which the entire electrode material takes part in the charging and discharging process^26^. Thus, explaining high values of specific capacitance. However, at high values of current density, the diffusion of ions into the whole electrode material is not possible due to the diffusion effect. As a result, the charging and discharging process only takes place at the electrode surface, which decreases the specific capacitance. The IR drop in the GCD curves is due to the resistance offered by the active electrode material, electrolyte, and the interface between electrode and electrolyte. The IR drop cannot be avoided in the supercapacitors. The specific capacitance reduces from 366.22 to 288.88 F g^−1^ as the current density changes from 4 to 100 A g^−1^. The electrode shows good capacitive behavior and high specific capacitance retention (79%) as the current density increases. Mostly, the specific capacitance of carbon is around 300 to 400 F g^−1^ and the specific capacitance of the fabricated thin film electrode is in good agreement with the expected values^30^.

The electrical response of the thin film electrode is studied using a non-destructive EIS characterization technique. The EIS measurements were carried in the 0.01 to 10^5^ Hz frequency range and at 5 mV AC perturbation. Figure 4c represents the Nyquist plot obtained from the EIS results and the intercept along Z´ gives the value of bulk resistance (R_b_). The R_b_ is the total resistance offered by the electrolyte, active material on the electrode, and resistance due to the interface between active material and substrate^31^. The value of R_b_ obtained from Fig. 4c is 1 Ω, which reveals good conductivity of the electrode^32^. Moreover, the Bode phase angle plot for the electrode is also shown in Fig. S4 (supplementary information). To investigate the use of carbon thin film electrode for supercapacitor applications, cyclic stability studies were performed at 0.06 A (120 A g^−1^). The cyclic stability shown in Fig. 4d illustrates that for 8000 cycles, the electrode retains 100% of its capacitance. Efficient cyclic stability can be explained due to an increase in the wettability of the electrode, which helps in maintaining the specific capacitance over various cycles^33^.

The carbon thin film electrode prepared from the waste facial mask has shown good electrochemical properties and can be used as a supercapacitor electrode. The comparison of the current work with previously reported works with 6.0 M KOH as an electrolyte is shown in Table 1. The comparison study reveals that the current work reports carbon thin film electrode with better stability and higher specific capacitance.

**Table 1:** Comparison of the electrochemical performance of the fabricated carbon thin film electrode with other reported works.

Waste material	Specific capacitance (F g^−1^)	Cyclic stability	Ref.
Coconut shell	228 at 5 mV s^−1^	93% after 3000 cycles	^34^
Watermelon rind	333.4 at 1 A g^−1^	96.8% after 10,000 cycles	^35^
Tea waste buds	332 at 1 A g^−1^	97.8% after 100,000 cycles	^36^
Withered rose flower	350 at 1 A g^−1^	96.5% after 15,000 cycles	^37^
Plastic waste (polyethylene terephthalate)	169 at 0.2 A g^−1^	90.6% after 5000 cycles	^38^
Mangosteen peels	357 at 1 A g^−1^	94.5% after 130,000 cycles	^39^
Shrimp shells	175 at 0.5 A g^−1^	94%, 1000 cycles	^40^
Cigarette filter	153.8 at 1 A g^−1^	Slight increase, 6000 cycles	^41^
Rose	208 at 0.5 A g^−1^	99%, 25,000 cycles	^42^
Bamboo fiber	258 at 0.1 A g^−1^	92% after 3000 cycles	^43^
Bamboo shoot	209 at 0.5 A g^−1^	95% after 10,000 cycles	^44^
Tree bark	236 at 0.5 A g^−1^	70.8% after 5000 cycles	^45^
Ginkgo leaves	323 at 0.5 A g^−1^	92.7% after 12,000 cycles	^46^
Polyethylene	100 at 0.5 A g^−1^	97.1% after 10,000 cycles	^47^
Polystyrene	208 at 1 A g^−1^	94.3% after 5000 cycles	^48^
Face mask	328.9 at 1 A g^−1^	81.1% after 3000 cycles	^49^
Face mask	366.2 at 4 A g^−1^	100% after 8000 cycles	**Current work**

#### NiO thin film electrode

**Figure 5:**
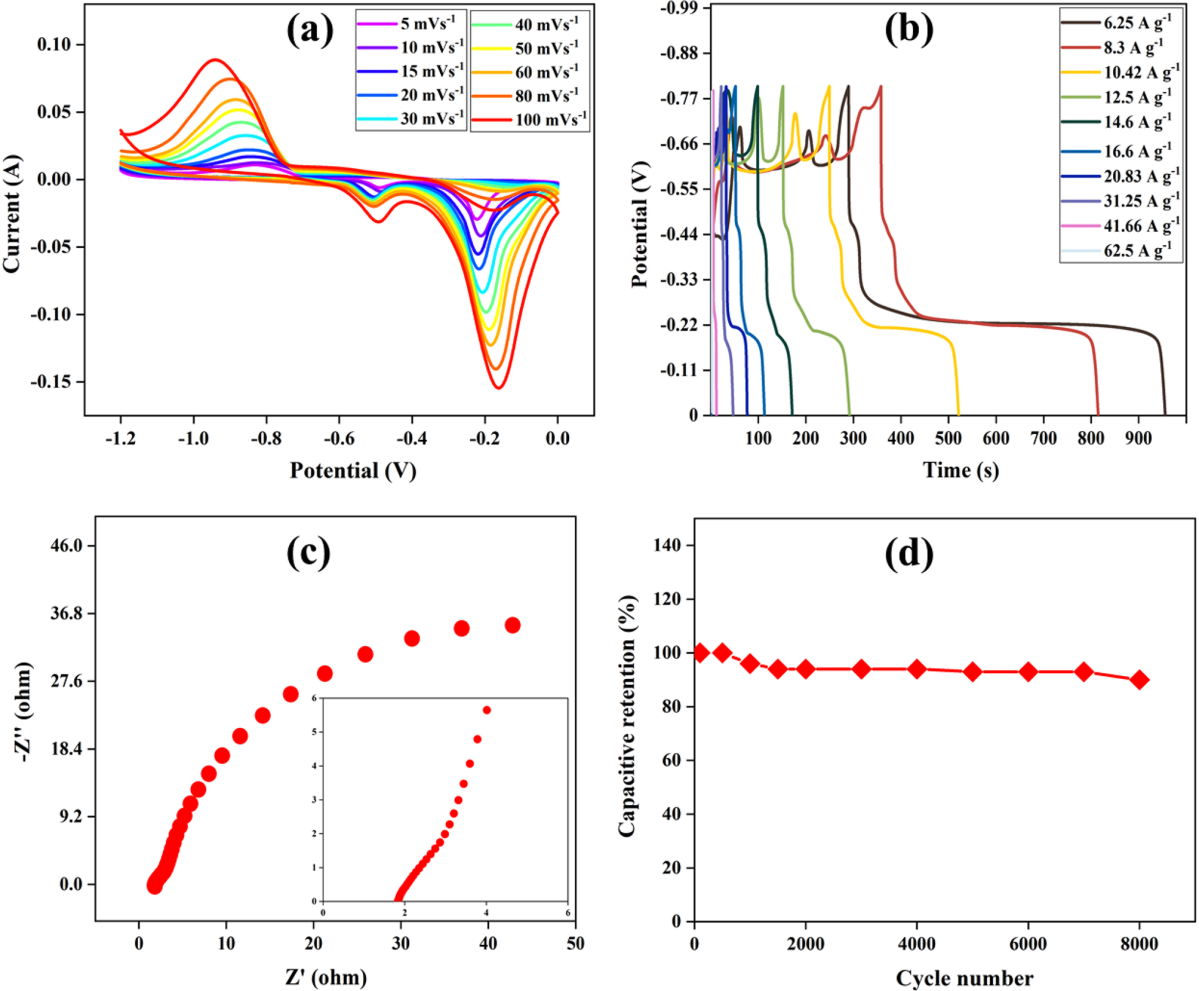
Electrochemical characterization of NiO thin film electrode. (**a**) CV at 5 to 100 mV s^−1^, (**b**) GCD at 6.25 to 62.5 A g^−1^, (**c**) EIS, and (**d**) cyclic stability.

The electrochemical performance of NiO thin film electrode is analyzed using CV, GCD, and EIS characterization. The CV curves in Fig. 5a are obtained at 5 to 100 mV s^−1^ of scan rate and − 1.2 to 0 V of potential range. The capacitance of the NiO thin film electrode again follows the redox mechanism as suggested by prominent redox peaks. With an increase in scan rate, the peak current increases, which is due to the highly capacitive nature and rapid electron and ion transport mechanism. The thin film electrode acts as a negative electrode due to the presence of CV curves in the negative potential region. Using Eq. (1), the specific capacitance of the electrode calculated from the CV curves is represented in Table S2 (supplementary information). From Dunn’s method, the charge storage mechanism of the electrode is found to be diffusion-controlled (Fig. S5 (supplementary information)). Moreover, using Eq. (4), the *b* value is 0.7 for anodic peak currents and 0.5 for the cathodic peak currents (Fig. S6 (supplementary information)). This again confirms the diffusion-controlled charge storage mechanism of the electrode.

The GCD tests performed in − 0.8 to 0 V at various current density are shown in Fig. 5b. The shape of the GCD curve is due to the characteristics of the active electrode material. The deviation of the GCD curves from the normal shape is due to the battery-type behavior of the electrode^50,51^. Using *m* = 0.00048 g in Eq. (1), the values of specific capacitance are 4987.5, 4570, 3387.5, 2100, 1277, 1200, 1100, 900, 350, and 150 F g^−1^ at 6.25, 8.3, 10.4, 12.5, 14.6, 16.6, 20.83, 31.25, 41.66, and 62.5 A g^−1^, respectively. The IR drop in the GCD tests is high for the electrode which suggests that more resistance is offered by the setup and various electrodes with high IR drop have been reported with good supercapacitor applications^52,53^. The EIS measurement for NiO thin film electrode is carried in 6 molar KOH electrolyte at 5 mV for the frequency range of 1 to 10^5^ Hz. Figure 5c shows the Nyquist plot obtained from the results. The value of R_b_ obtained from the plot is 1.8 Ω, which shows that the electrode possesses good electrical conductivity. The bulk resistance of the NiO thin film electrode is higher than the carbon thin film electrode, which explains the high IR drop and more rapid decrease in the specific capacitance with increasing current density. The Bode phase angle plot of the electrode is shown in Fig. S7 (supplementary information). To test the performance of the electrode, the cyclic stability test shown in Fig. 5d is performed at 0.025 A (52.08 A g^−1^). The electrode is capable of retaining 95% of its specific capacitance for 8000 cycles, which shows that the electrode can be used for supercapacitor applications. The average specific capacitance of the fabricated NiO thin film electrode is approximately equal to its theoretical value^54^.

Therefore, the electrochemical analysis of both carbon and NiO thin film electrodes reveals that the electrodes possess good electrical performance suitable for supercapacitor applications.

## Supercapacitor applications

**Figure 6:**
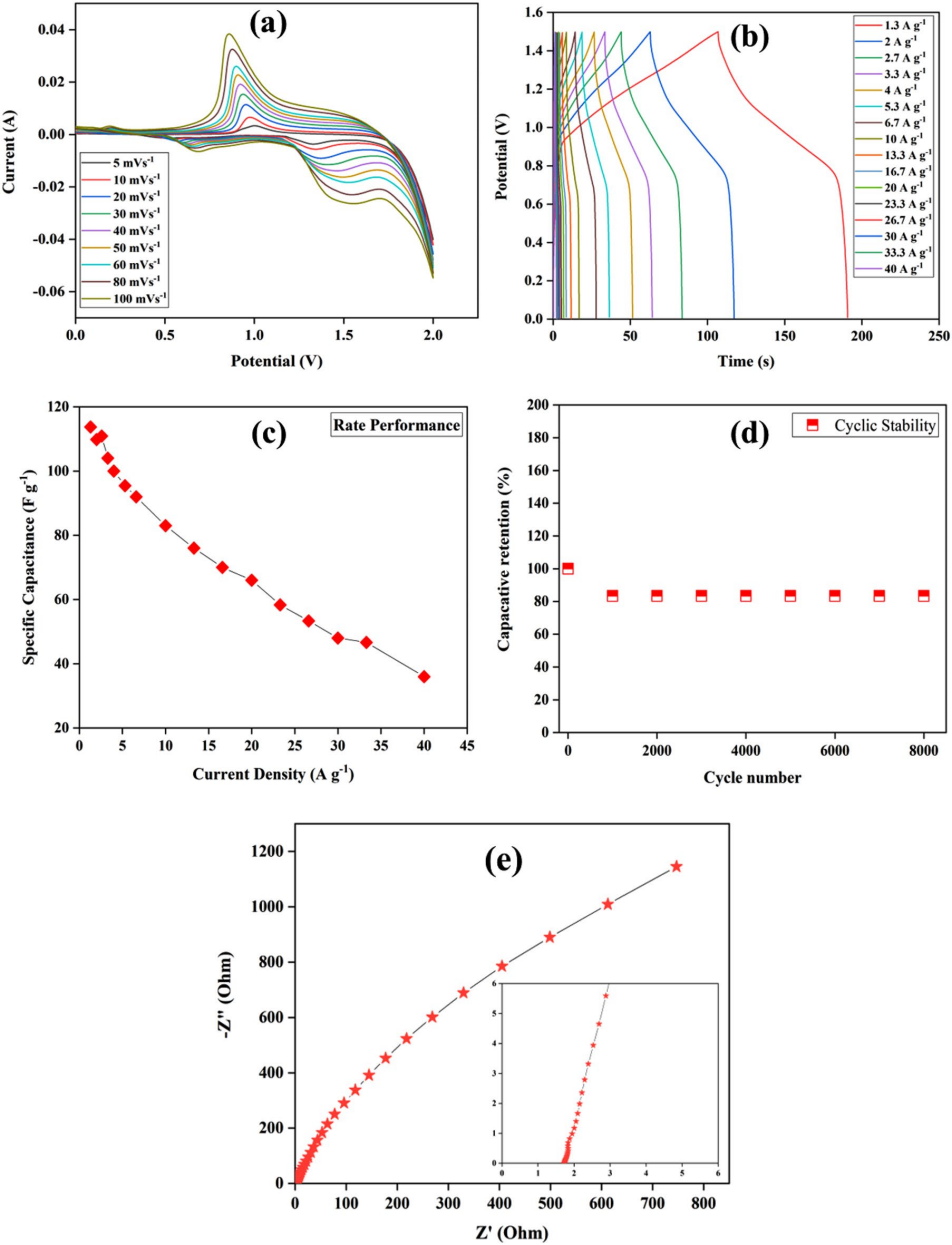
(**a**) CV study performed at 5 to 100 mV s^−1^, (**b**) GCD tests performed at 1.3 to 40 A g^−1^, (**c**) Rate performance, (**d**) cyclic stability, and (**e**) EIS study of the device.

For investigating the supercapacitor applications, a two-electrode set-up is used to perform the electrochemical characterization techniques. A 6.0 M KOH solution is utilized as an electrolyte whereas NiO thin film electrode is used as the working electrode and a carbon thin film is used as the counter electrode (short with reference electrode). The CV measurements are performed at a scan rate of 5 to 100 mV s^−1^ and − 1.4 to 0 V is taken as a potential range. The CV curves for the supercapacitor are shown in Fig. 6a. The specific capacitance of the device calculated using Eq. (1) is represented in Table S3 (Supplementary Information). As the scan rate increases, the peak current increases, which denotes that the capacitance of the supercapacitor follows the redox mechanism. Figure 6b shows the GCD curves obtained in 0 to 1.5 V of potential range at various current density. Using *m* = 0.001 g in Eq. (1), the specific capacitance is 113.73, 109.8, 110.93, 104, 100, 95.46, 92, 83, 76, 70, 66, 58.33, 53.33, 48, 46.66 and 36 F g^−1^ at 1.3, 2, 2.6, 3.3, 4, 5.3, 6.6, 10, 13.3, 16.6, 20, 23.3, 26.6, 30, 33.3, and 40 A g^−1^, respectively. The decrease in the specific capacitance with increasing current density can be explained due to the diffusion effect. The specific capacitance of the device decreases from 113.73 to 36 F g^−1^ as the current density increases from 1.3 to 40 A g^−1^ with a 68% reduction in the specific capacitance of the supercapacitor. The rate performance of the device is shown in Fig. 6c. The cyclic stability test (Fig. 6d) performed at 0.05 A for the supercapacitor shows 83% specific capacitance retention over 8000 cycles. The EIS measurement for the two-electrode supercapacitor is carried in 6.0 M KOH electrolyte at 5 mV for the frequency range of 1 to 10^5^ Hz. The corresponding Nyquist plot is shown in Fig. 6e. The value of R_b_ obtained from the plot is 1.73 Ω, which shows that the device has good electrical conductivity. Moreover, the Bode phase angle plot for the device is shown in Fig. S8 (supplementary information).

**Figure 7:**
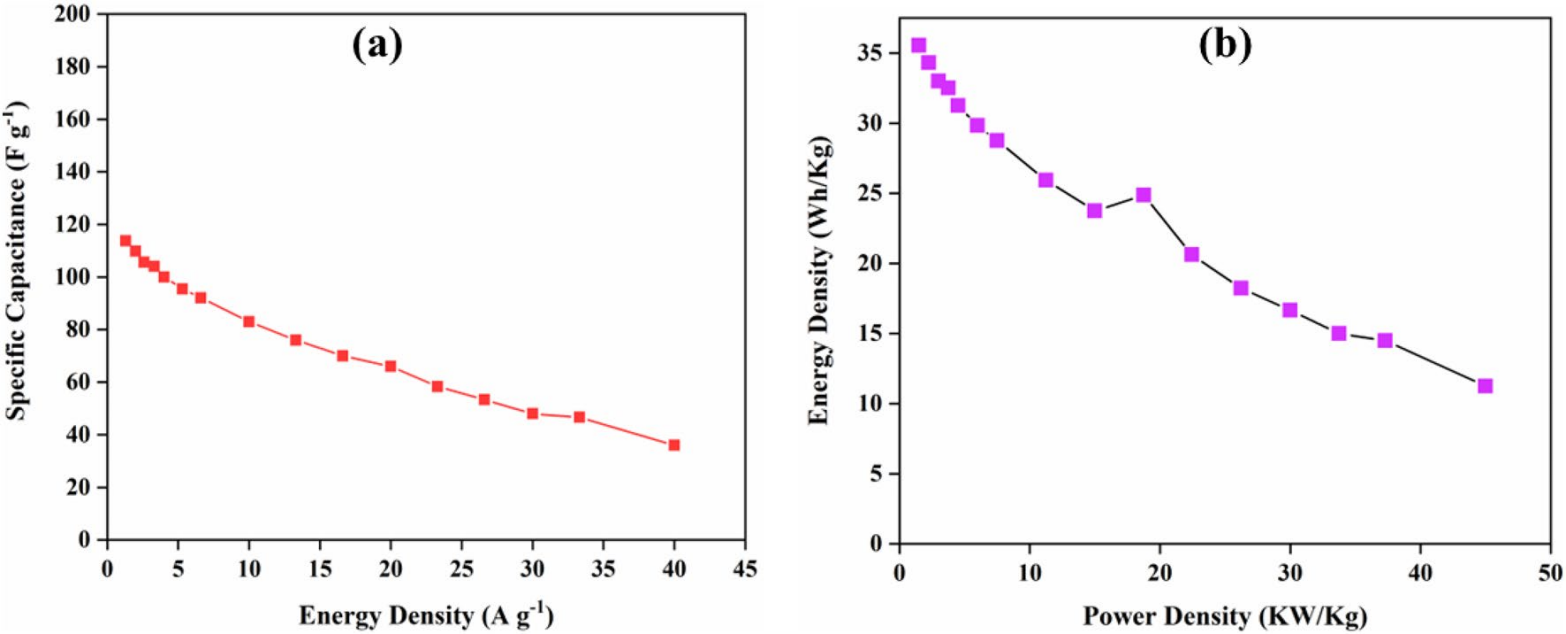
(**a**) Specific capacitance vs energy density and (**b**) Ragone’s plot of the supercapacitor device.

Using Eq. (3), the calculated values of energy density are 35.54, 34.31, 33, 32.5, 31.25, 29.83, 28.75, 25.93, 23.75, 21.87, 20.62, 18.22, 16.66, 15, 14.5, and 11.25 Wh Kg^−1^ at the current density of 1.3, 2, 2.6, 3.3, 4, 5.3, 6.6, 10, 13.3, 16.6, 20, 23.3, 26.6, 30, 33.3, and 40 A g^−1^, respectively. The graph between specific capacitance and energy density is shown in Fig. 7a. Moreover, the power density values are 1.5, 2.25, 3, 3.75, 4.5, 6, 7.5, 11.25, 15, 18.75, 22.45, 26.24, 30, 33.75, 37.3, and 45 KW Kg^−1^ for the similar current density. A graph between the energy density and power density called the Ragone plot for the supercapacitor device is shown in Fig. 7b. The Ragone plot depicts that there is a decrease in the energy density with increasing power density, which suggests that the device can be used as a supercapacitor^55^.

The practical application of the supercapacitor device with fabricated electrodes is also carried out in this work. The supercapacitor device is prepared with a positive carbon thin film electrode, a negative NiO thin film electrode, and a KOH separator. The KOH separator is prepared by mixing KOH and PVA in a particular ratio in DI water. The solution is then subjected to heat treatment and continue stirring until a clear solution is obtained. The solution is then poured into a petri dish and left for overnight drying, forming a KOH separator. The separator is then placed in between the two electrodes and the device is held using pins. The separator is placed such that the two electrodes should not touch each other which otherwise will result in short-circuiting the device. The prepared supercapacitor device is shown in Fig. S9 (Supplementary Information).

**Figure 8:**
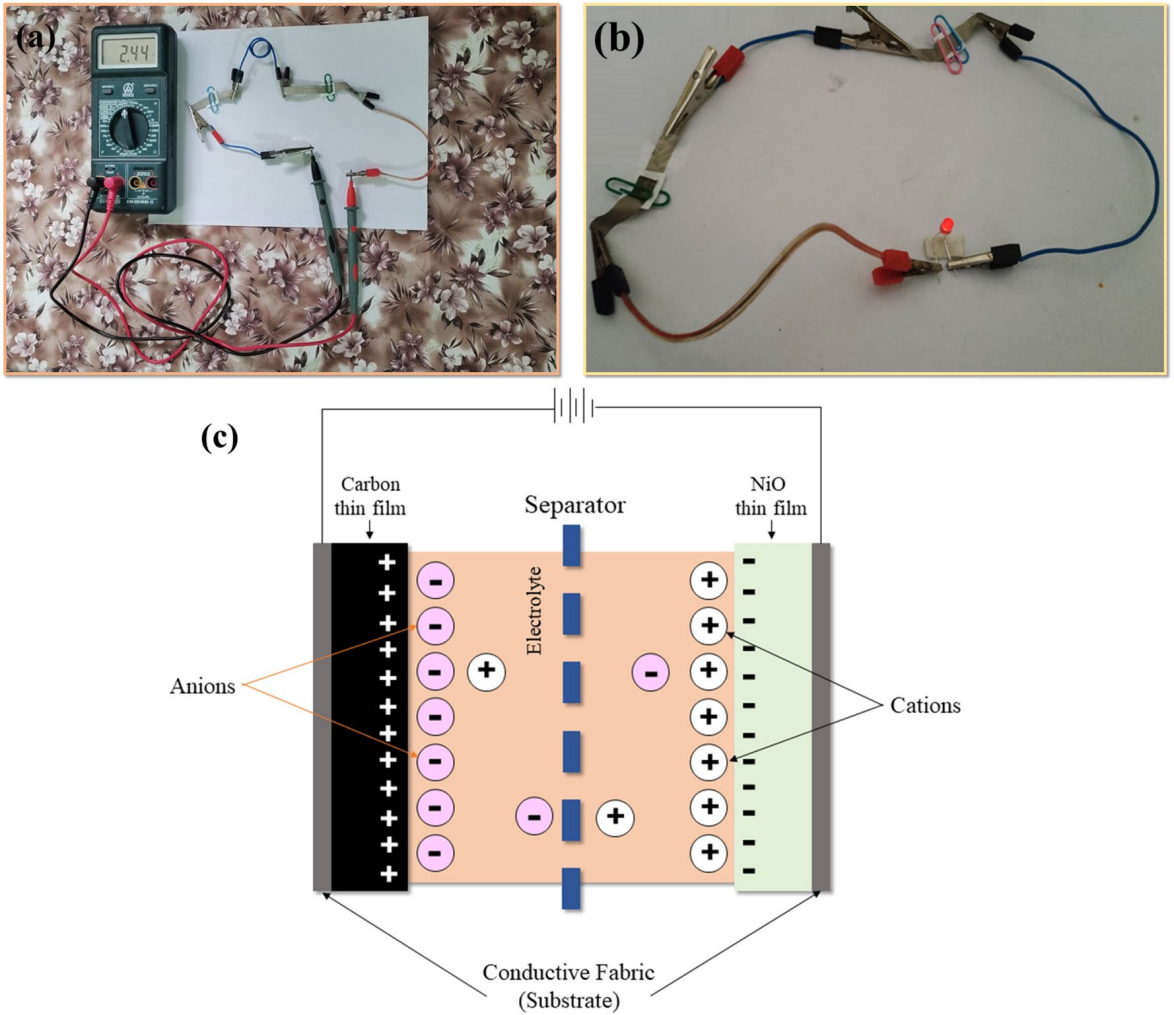
(**a**) Practical demonstration with the multimeter, (**b**) glowing red LED, and (**c**) working mechanism of the supercapacitor.

Two supercapacitor devices were connected in series and charged using a battery. The battery is then removed and a multimeter is connected across the combination to measure the potential. The potential across the combination is around 2.5 V as shown in Fig. 8a and the video is also recorded for the measurement. Similarly, the combination is again charged using a battery, and a red LED was connected across the terminals after removing the battery. The combination can glow the LED as shown in Fig. 8b and the video is again recorded for the practical demonstration. The red LED is successfully functional for 6 min with a full glow for 4 min i.e., the intensity starts to decrease after 4 min. The practical demonstration successfully advocates the application of fabricated carbon and NiO thin film electrodes for supercapacitor application.

The mechanism governing the working of the supercapacitor device is diagrammatically shown in Fig. 8c. The KOH electrolyte present contains both positive and negative charge ions i.e., K^+^ and OH^−^. During the charging process, the carbon thin film electrode develops a positive charge and NiO thin film electrode develops a negative charge. As a result, the K^+^ ions move toward NiO thin film electrode whereas OH^−^ ions move toward the carbon thin film electrode. Hence, a thin layer of ions is developed on the inner side of both electrodes leading to the formation of the electrostatic double layer. Thus, a single supercapacitor device is comparable to a series combination of two capacitors, which explains the high specific capacitance of a supercapacitor as compared to capacitors. Similarly, the discharging process is the reverse of the charging process.

Further, the *b* value for both thin film electrodes is close to 0.5, which suggests that the charging and discharging are governed by the diffusion process. The value of R_b_ for both the electrodes is low i.e., 1.0 and 1.8 Ω, suggesting good conductivity of the electrodes. This helps in the smooth charging and discharging of the device.

## Conclusion

In this work, the upcycling of the surgical facial mask waste produced during the COVID-19 pandemic into the carbon thin film supercapacitor electrode is reported. The carbon thin film is fabricated by preparing a slurry paste and depositing it on a wearable conductive fabric. The electrochemical performance is analyzed in a 6 M KOH electrolyte. The carbon thin film acts as a positive electrode with a specific capacitance of 366.2 F g^−1^ at 2 A g^−1^. The carbon thin film electrode shows good cyclic stability with 100% specific capacitance retention for 8000 cycles. A separate NiO thin film electrode is prepared to be used as a negative electrode having 4987.5 F g^−1^ specific capacitance at 6.25 A g^−1^. The NiO thin film electrode shows 95% specific capacitance retention after 8000 cycles. The supercapacitor device with NiO thin film as the working electrode, carbon thin film as the counter electrode (reference electrode being short), and 6 M KOH as an electrolyte, displays 113.73 F g^−1^ of specific capacitance at 1.3 A g^−1^ current density. The supercapacitor retains 83% of its specific capacitance for 8000 cycles during the cyclic stability test. The supercapacitor device with the fabricated electrodes and KOH separator glows a red LED for 6 min. The work aims to advance sustainable development by attempting to mitigate the adverse effect of facial mask waste disposal on the environment.

## Supplementary Information

Supplementary Information.

## Data Availability

The raw/processed data required to reproduce these findings will be available on the reasonable request.
