# Supplementary material for: Upcycling of surgical facemasks into carbon based thin film electrode for supercapacitor technology
Source: Sci Rep. 2023 Jul 27;13:12146. doi: 10.1038/s41598-023-37499-x (PMC10374911; doi:10.1038/s41598-023-37499-x)
Supplement: Supplementary file 1 — Supplementary Information. [file 41598_2023_37499_MOESM1_ESM.doc]

**Supplementary Information**

**Upcycling of Surgical Facemasks into Carbon based Thin Film Electrode for Supercapacitor Technology**

Aamir Ahmed^1^, Sonali Verma^1^, Prerna Mahajan^1^, Ashok K. Sundramoorthy^2^, Sandeep Arya^1,*^

^1^Department of Physics, University of Jammu, Jammu, Jammu and Kashmir- 180006, India

^2^Centre for Nano-Biosensors, Department of Prosthodontics, Saveetha Dental College and Hospitals, Saveetha Institute of Medical and Technical Sciences, Chennai 600077, Tamil Nadu, India

^*^Corresponding author email: [snp09arya@gmail.com](mailto:snp09arya@gmail.com)


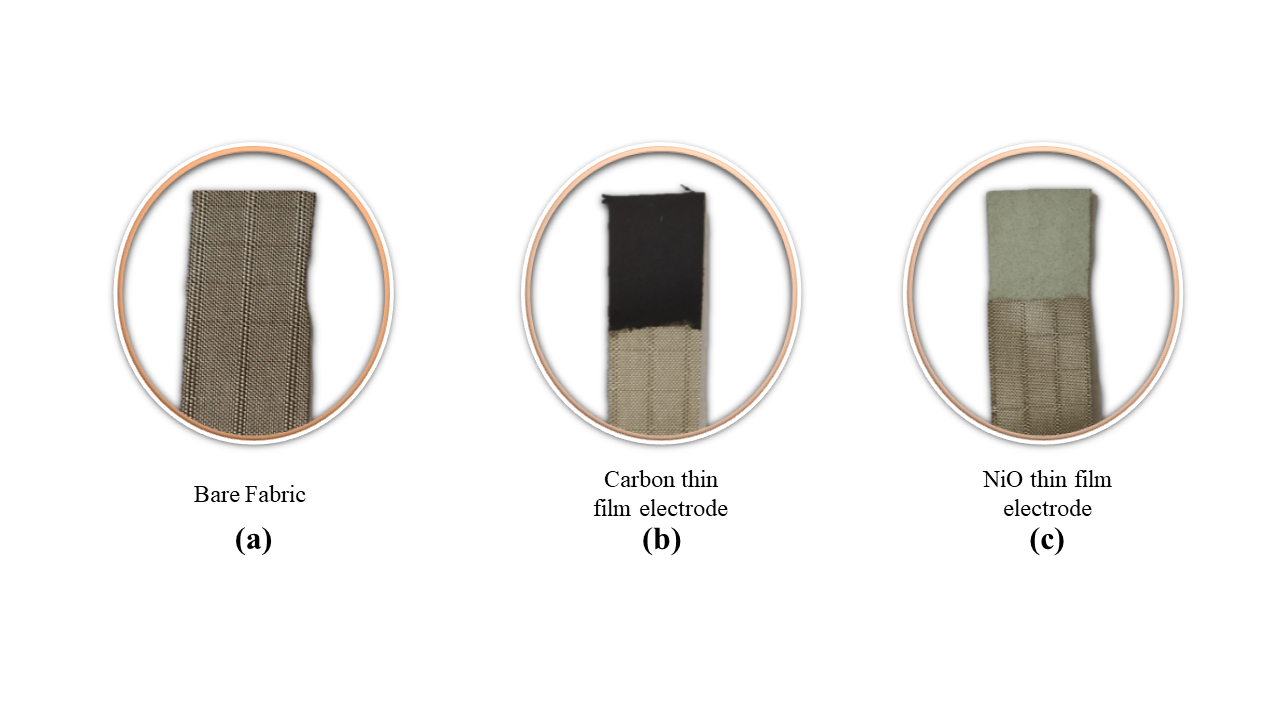


**Figure S:** (a) Bare fabric, (b) carbon thin film electrode, and (c) NiO thin film electrode.


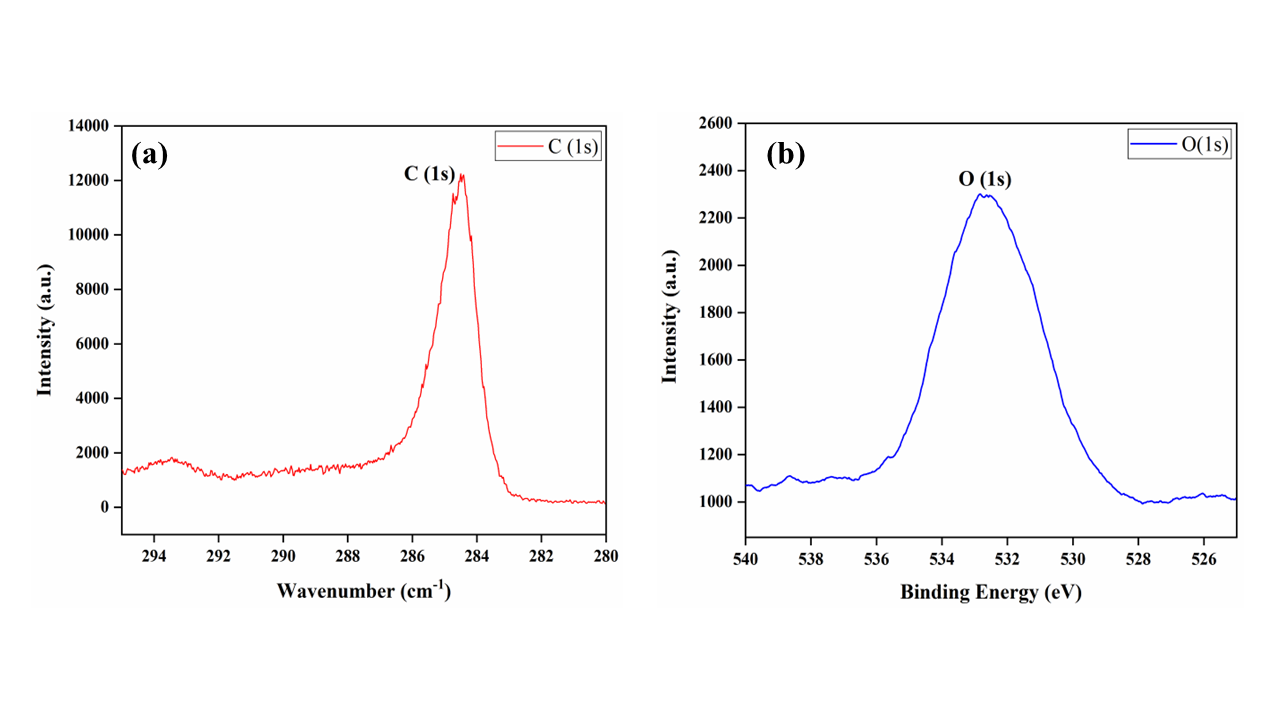


**Figure S1**: High resolution XPS spectra of (a) C 1s and (b) O 1s.

**Table S1**: Specific capacitance of carbon thin film electrode from CV curves.

| **Scan rate (mV s^-1^)** | **Specific Capacitance ( F g^-1^)** |
| --- | --- |
| 5 | 733.33 |
| 10 | 700 |
| 20 | 666.66 |
| 30 | 632.55 |
| 40 | 605.33 |
| 50 | 600 |
| 60 | 555.55 |
| 80 | 541.66 |
| 100 | 533.33 |
| 200 | 500 |


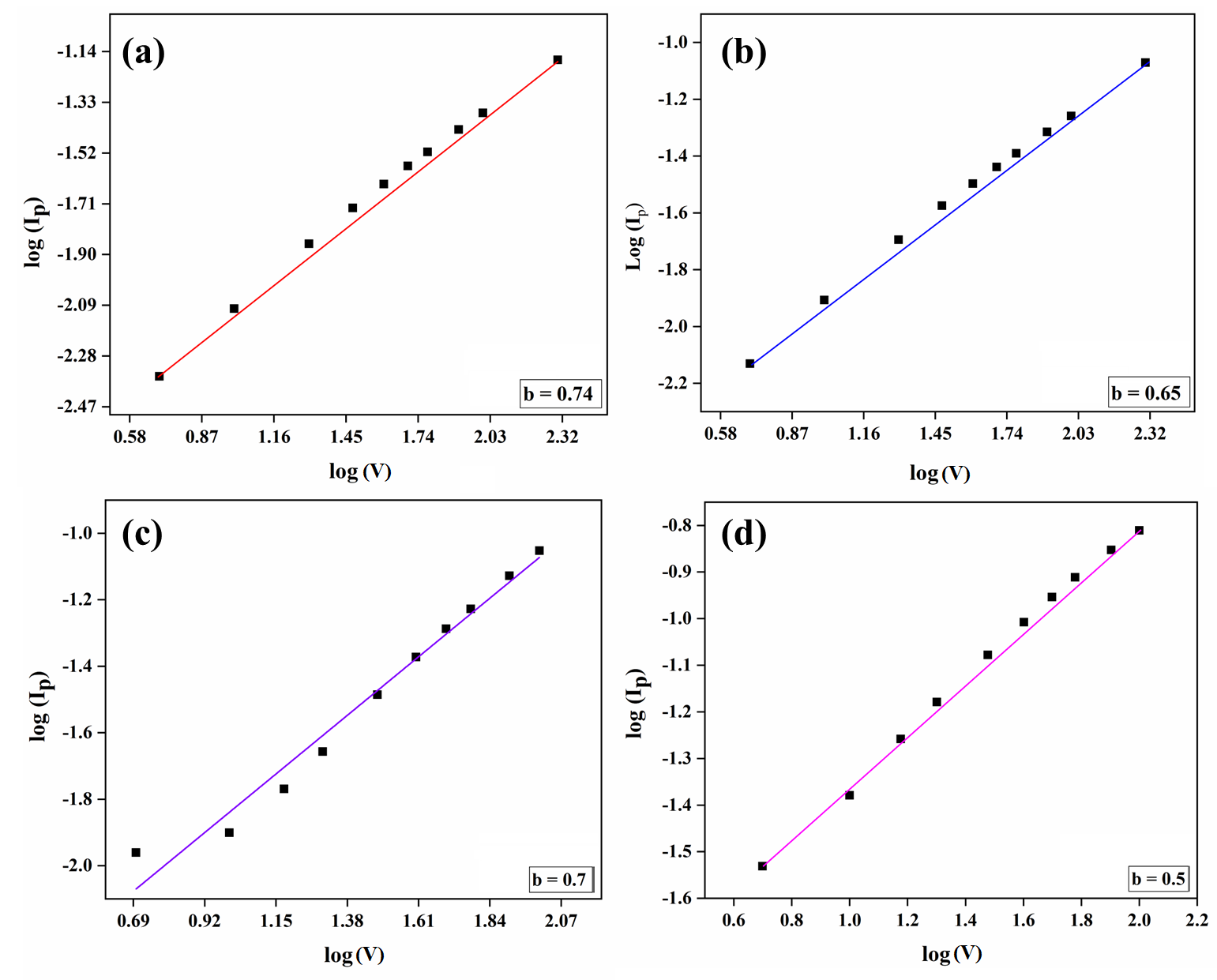


**Figure S2**: Value of *b* for (a) anodic peak currents and (b) cathodic peak currents of carbon thin film electrode.


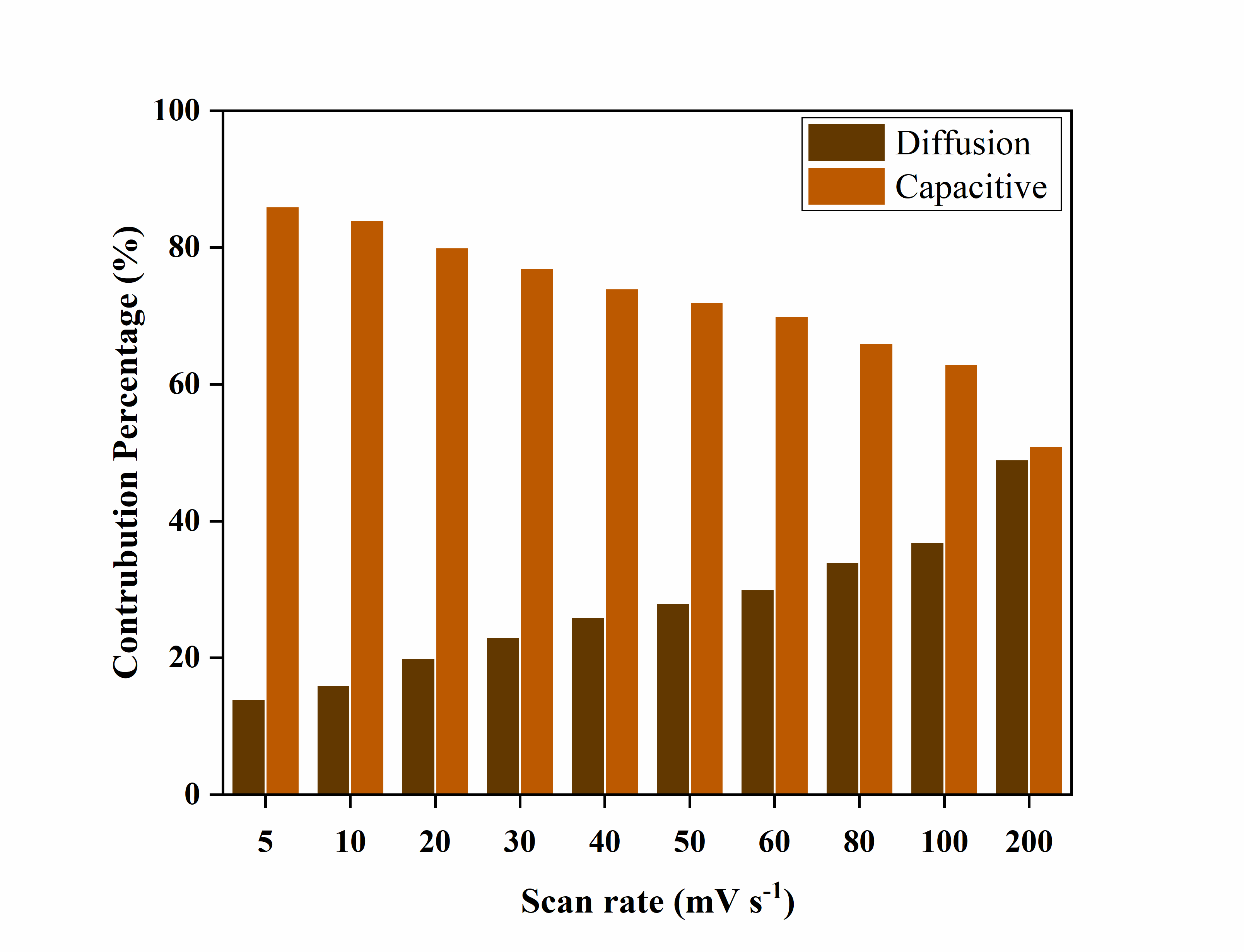


**Figure S3**: Diffusion and capacitive contribution of the carbon thin film electrode.


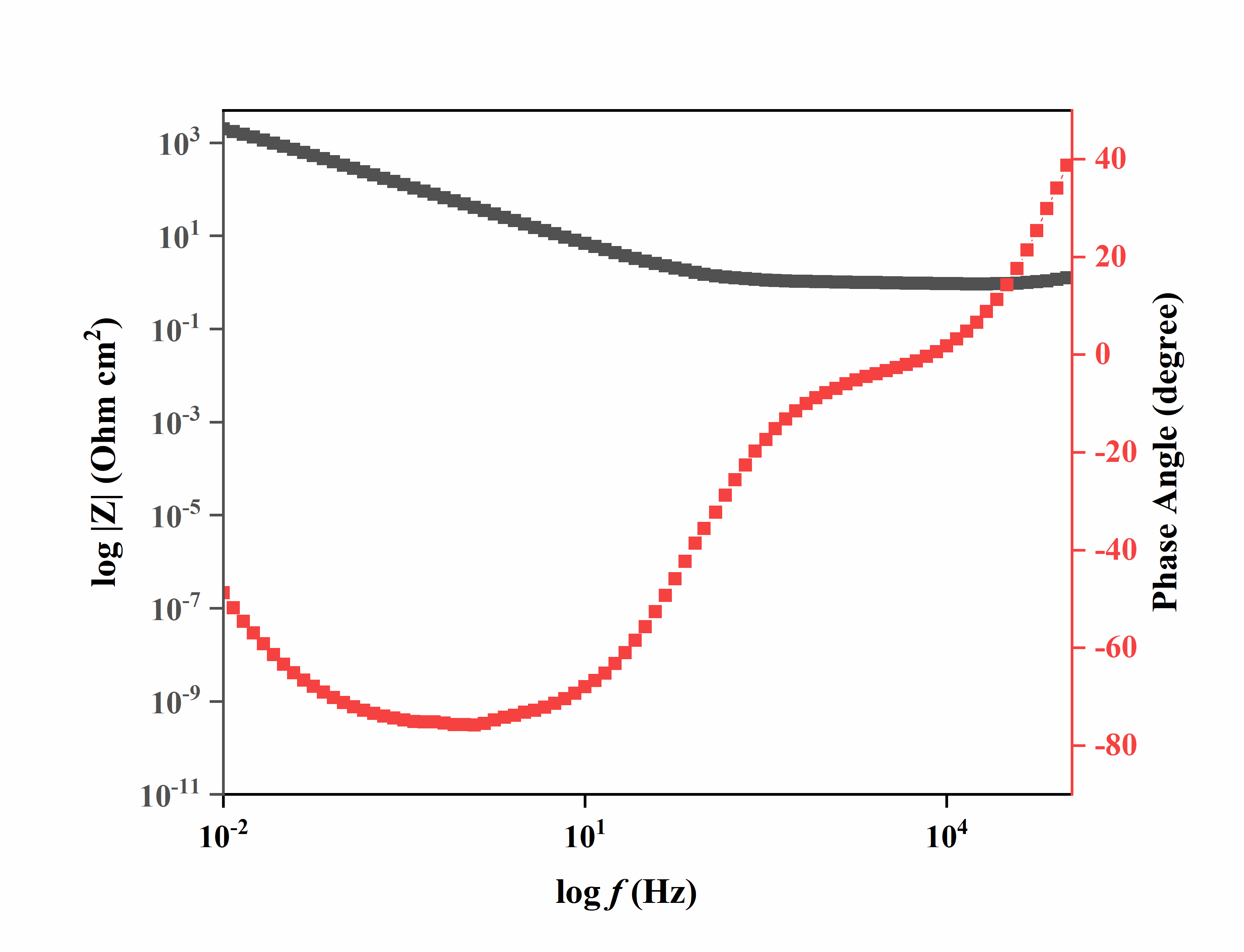


**Figure S4**: Bode phase angle plot for carbon thin film electrode.

**Table S2**: Specific capacitance of NiO thin film electrode from CV curves.

| **Scan rate (mV s^-1^)** | **Specific Capacitance ( F g^-1^)** |
| --- | --- |
| 5 | 2430.57 |
| 10 | 1736.11 |
| 15 | 1504.63 |
| 20 | 1388.89 |
| 30 | 1257.40 |
| 40 | 1171.87 |
| 50 | 1111.11 |
| 60 | 1041.66 |
| 80 | 976.56 |
| 100 | 868.05 |


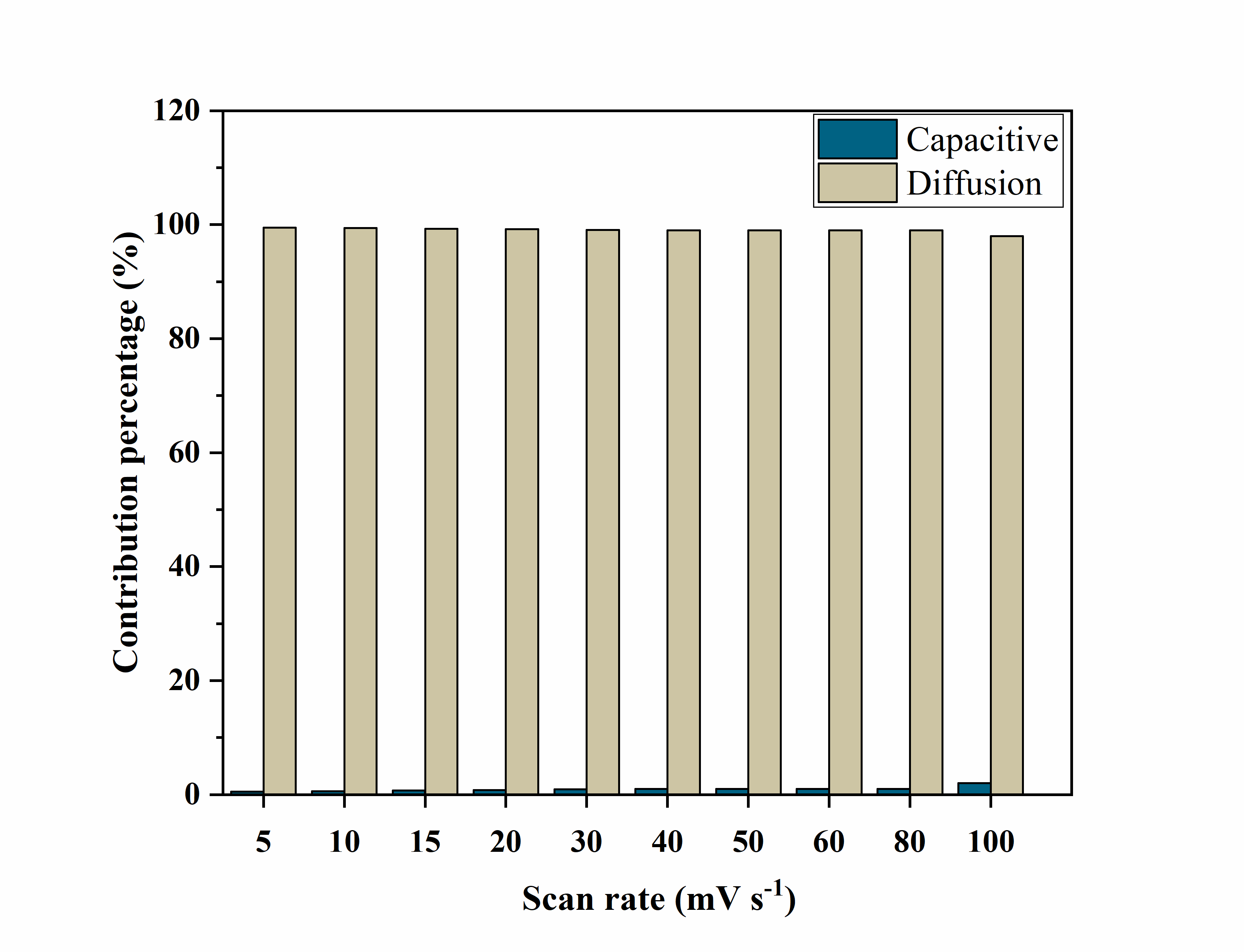


**Figure S5**: Capacitive and diffusion contribution of NiO thin film electrode.


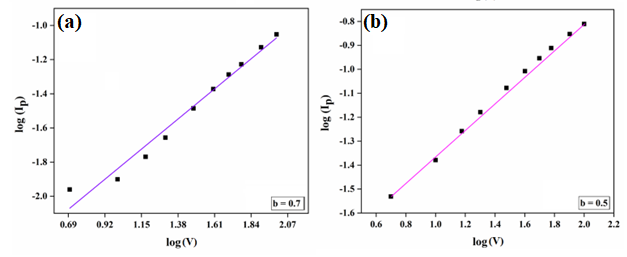


**Figure S6**: Value of *b* for (a) anodic peak currents and (b) cathodic peak currents of NiO thin film electrode.


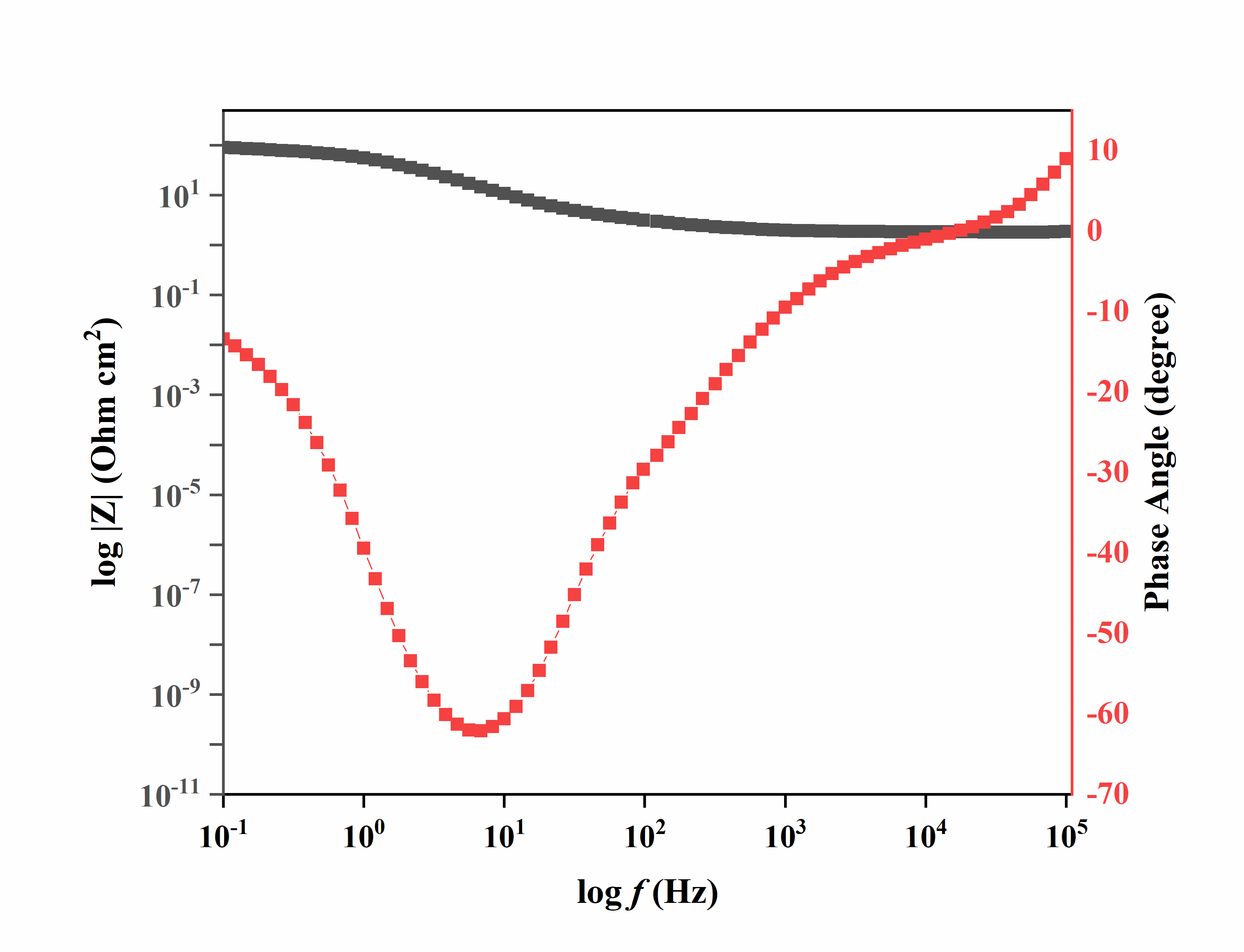


**Figure S7**: Bode phase angle plot of NiO thin film electrode.

**Table S3: Specific capacitance of the device calculated from CV curves.**

| **Scan rate (mV s^-1^)** | **Specific Capacitance ( F g^-1^)** |
| --- | --- |
| 5 | 350 |
| 10 | 280 |
| 20 | 232.50 |
| 30 | 206.66 |
| 40 | 193.75 |
| 50 | 186 |
| 60 | 179.16 |
| 80 | 172.5 |
| 100 | 163.5 |
| 5 | 350 |


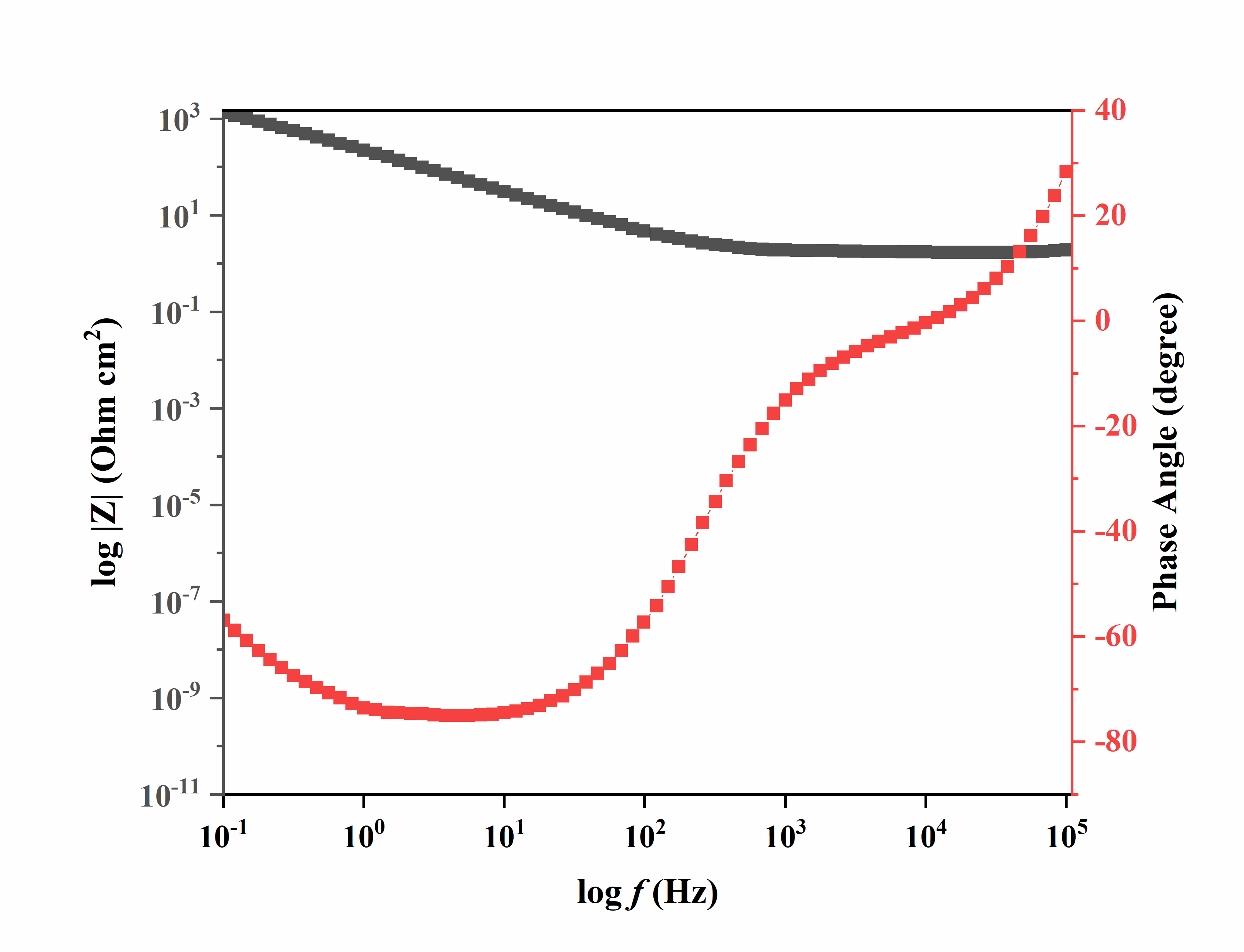


**Figure S8:** Bode phase angle plot of the supercapacitor device.


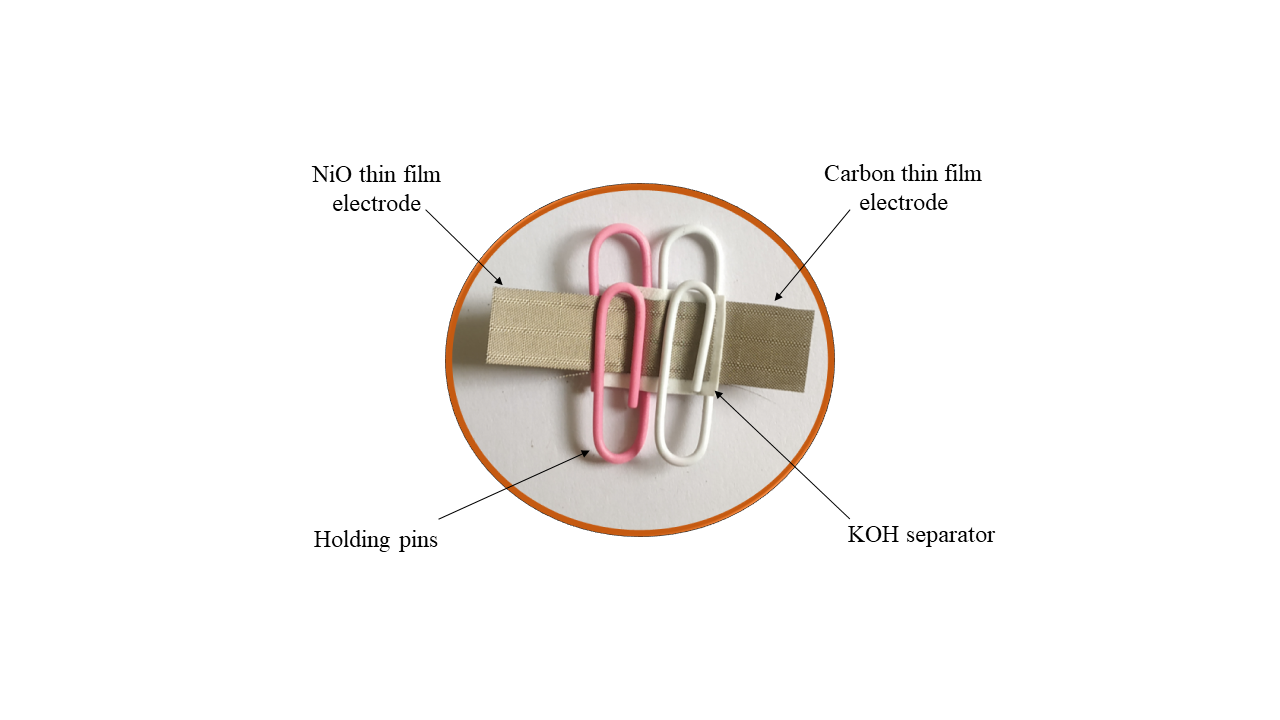


**Figure S9**: Image of carbon and NiO thin film supercapacitor device.
